# Characterization of the profile and clinical variables associated with mortality in a Brazilian coronary ICU

**DOI:** 10.1186/cc13379

**Published:** 2014-03-17

**Authors:** CE Bosso, VF Paula, OA Souza, GE Valerio, SV Ferreira

**Affiliations:** 1 Instituto do Coração de Presidente Prudente, Brazil; 2 UNOESTE - Universidade do Oeste Paulista, Presidente Prudente, Brazil

## Introduction

Cardiovascular disease is the leading cause of death worldwide and the outlook for 2020 data is even more alarming [1],[2]. In this context, the coronary ICUs (CICUs) have become increasingly numerous. However, data concerning this are still very limited in the literature. This study aims to characterize the profile of CICU admissions in Brazil and the main clinical variables associated with increased mortality.

## Methods

To conduct the study we analyzed the database of a CICU of a medium-sized hospital in the city of Presidente Prudente, Brazil. Admissions that occurred during the period 1 September 2010 to 31 August 2013 were analyzed. The information was collected from the EPIMED MONITOR system and statistically analyzed using EPI INFO, version 3.5.2 software. *P *< 0.05 two-tailed was considered significant, and confidence intervals at 95% (95% CI) were used for the logistic regression multivariate estimated in the sample.

## Results

A total of 2,098 admissions were recorded, of which 42.1% were female and 57.9% male. The average age was 66.99 ± 13.30 years. The prognosis for SAPS 3 admission score averaged 40.8 ± 15.48 points and the mean unit length of stay was 3.5 ± 5.01 days. The main admission diagnoses were unstable angina (13.06%), non-ST-segment elevation myocardial infarction (8.29%), ST-segment elevation myocardial infarction (8.10%) and supraventricular cardiac tachyarrhythmia (6.91%). We observed a higher risk of death among patients who had, at admission, congestive heart failure NYHA 2, 3 or 4 (odds ratio (OR) = 2.81, 95% CI: 2.07 to 3.83, *P *= 0.001), chronic renal failure (OR = 2.48, 95% CI: 1.68 to 3.67, *P *= 0.001), peripheral artery disease (OR = 1.93, 95% CI: 1.04 to 3.58, *P *= 0.033), severe chronic obstructive pulmonary disease (OR = 3.17, 95% CI: 1.82 to 5.51, *P *= 0.002), and infection at admission (OR = 7.88, 95% CI: 5.27 to 11.78, *P *= 0.001).

## Conclusion

The results reported may direct healthcare professionals to profile the patient hospitalized in a CICU, paying attention to the most disturbing and lethal comorbidities present on admission.
